# Acute Rheumatic Fever Beyond Childhood: Case Series of Adult Recurrence and Atypical Manifestations

**DOI:** 10.7759/cureus.103958

**Published:** 2026-02-20

**Authors:** Sandhya S, Dany P John, Senthilnathan Pichaipillai, John Jose, Paul V George

**Affiliations:** 1 Cardiology, Christian Medical College, Vellore, IND

**Keywords:** aso, carditis, mitral stenosis, penicillin prophylaxis, rheumatic fever

## Abstract

Acute rheumatic fever (ARF) is an autoimmune condition following a preceding beta-haemolytic streptococcal throat infection. It affects children in the age group of 5-15 years predominantly. In the older age group, the incidence decreases, with the first episode being very rare, though recurrence may occur. Further, patients who develop ARF in childhood, unless they quit the penicillin prophylaxis, recurrence or reactivation is also less.

This is a retrospective case series of six adult patients aged >30 years who presented to our hospital in the last one year and were diagnosed with either ARF or reactivation as per the 2015 revised Jones criteria.

This study was conducted to emphasise that ARF is not an uncommon disease in adults in our country. Though the patient may have subtle symptoms and signs, we need to identify them and evaluate further with the required investigations to prove the diagnosis with the modified Jones criteria.

When an adult patient presents with fever, joint pain, and valvular lesions, rheumatic fever is least thought of due to the age of the patient, and multiple other differentials are considered. Patients in our case series had varied presentations and acute rheumatic activity. To conclude, physicians should have a low threshold to consider ARF irrespective of age, and once ARF is diagnosed, patients need to be properly counselled about the strict adherence to penicillin prophylaxis.

## Introduction

Acute rheumatic fever: background and epidemiology

Acute rheumatic fever (ARF) is an autoimmune condition following a preceding beta-haemolytic streptococcal throat infection. It affects children in the age group of 5-15 years predominantly. It is a major health problem in many countries affecting children and adolescents [1,2]. In the older age group, the incidence decreases, with the first episode being very rare, though recurrence may occur. It is a universal fact that failure to adhere to penicillin prophylaxis is an important determinant for rheumatic fever recurrence [3]. The recurrences are common in young people and within five years of the first episode. Recurrence of ARF is also a risk factor for severe chronic valvular diseases [4].

The diagnostic challenge in adults

Due to its strong association with the paediatric age group, ARF is often under-recognised or missed in adult patients [5]. When an adult presents with non-specific symptoms, such as fever, polyarthritis, or new/worsening valvular lesions, the diagnosis of ARF is often the least considered differential. This diagnostic challenge is compounded by the fact that adult cases often present with subtle or atypical signs and symptoms [6,7], necessitating a high index of suspicion and thorough investigation using the modified Jones criteria. Additionally, some adult patients with pre-existing valvular disease may discontinue their secondary penicillin prophylaxis, leading to preventable recurrence.

The aim of this case series

Despite the perceived rarity, ARF is not an uncommon disease in the adult population within our country, with a prevalence rate of 0.2 per 1000 population [8]. To emphasise the need for heightened awareness, this case series collects and presents the clinical, laboratory, and echocardiographic details of patients over 30 years of age who presented to our institution over the past one year with acute ARF episodes or recurrences. First episode of ARF was diagnosed when the patient had no previous history of ARF, mitral stenosis (MS), and/or aortic stenosis (AS), and recurrence was diagnosed when the patient had previous ARF, MS, and/or AS based on the Jones criteria. We aim to explore the patients’ details such that these interpretations help us to make an early diagnosis of the first ARF and recurrences.

## Materials and methods

In this case series, we retrospectively analysed adult patients (more than 30 years) who presented to the cardiology outpatient department, and patients who were admitted to the cardiology ward in the past one year with atypical presentations of rheumatic fever (such as sole presentation of cardiac failure, fever of unknown origin, and prolonged illness), and patients of chronic rheumatic heart disease (RHD) with ARF recurrence, as identified in our electronic medical records. These patients were ultimately diagnosed with either ARF/reactivation based on the 2015 Revised Jones criteria, using a combination of antistreptococcal serology, clinical, and echocardiographic data. Patients less than 30 years of age and who did not meet the 2015 revised Jones criteria were excluded.

Demographic features, history, clinical details, ECG, echocardiography findings, and laboratory values for antistreptococcal titres and inflammatory markers were studied, and an analysis was made.

## Results

Over a one-year duration, six adult patients were identified who fulfilled our inclusion criteria, presenting with atypical symptoms and confirmed ARF/reactivation according to the 2015 Revised Jones criteria. Out of six patients, three were diagnosed to have reactivation and three had first episode of ARF (Table 1).

**Table 1:** Patient details Non-compliant means to be irregular on penicillin prophylaxis, and compliant means to be regular on penicillin prophylaxis. AVR, aortic valve replacement; DVR, double valve replacement.

Case no	Type	Carditis	Arthritis	Prophylaxis	Outcome	Follow-up
1	Reactivation	No	Yes	Non-compliant	Resolved, on regular penicillin prophylaxis	Remains asymptomatic on six months of follow-up
2	First episode	Yes	No	Not applicable	Resolved, on regular penicillin prophylaxis and planned for AVR	Remains clinically stable on four months of follow-up, awaiting AVR
3	First episode	Yes	Yes	Not applicable	Resolved, on regular penicillin prophylaxis and planned for DVR	Remains clinically stable on 10 months of follow-up, awaiting DVR
4	Reactivation	Yes	No	Non-compliant	Resolved, on regular penicillin prophylaxis	Remains asymptomatic on one year of follow-up
5	Reactivation	No	Yes	Compliant	Started on a twice-weekly regimen	Remains asymptomatic on three months of follow-up
6	First episode	Yes	No	Not applicable	Resolved, on regular penicillin prophylaxis	Remains clinically stable on nine months of follow-up

Case 1

Recurrence and Non-adherence in a 37-Year-Old

Presentation: A 37-year-old female from Chhattisgarh, previously diagnosed with RHD presenting as moderate MS and severe mitral regurgitation (MR) one year before, presented with a two-month history of migratory polyarthralgia involving multiple large joints. Crucially, she reported non-adherence to secondary penicillin prophylaxis since her RHD diagnosis. She denied fever, chest pain, or dyspnoea.

Clinical findings: Physical examination revealed a hyperdynamic apex, soft S1, with an apical mid-diastolic murmur and a pansystolic murmur. The electrocardiogram (ECG) showed sinus rhythm with a normal PR interval of 160 ms. Echocardiography confirmed the known valvular pathology: moderate MS (mitral valve area: 1.3 cm^2 ^and mean pressure gradient: 9 mmHg) and moderate MR, with normal left ventricular function.

Laboratory and diagnosis: Active inflammation was confirmed with significantly elevated C-reactive protein (CRP: 46 mg/L) and erythrocyte sedimentation rate (ESR: 120 mm in one hour). Streptococcal titres were markedly elevated: anti-streptolysin O (ASO: 1520 IU/mL) and anti-DNase B (ADNB: 2800 U/mL) (Table 1). The patient met the modified 2015 Jones criteria for ARF recurrence (polyarthritis and evidence of recent streptococcal infection).

Management and outcome: She was immediately initiated on penicillin and treated with naproxen for six weeks [9], leading to complete resolution of her arthritis. Following extensive counselling, she was discharged with a strict regimen for long-term secondary penicillin prophylaxis.

Case 2

Acute Reactivation Presenting With Decompensated Valvular Disease in a Patient With Coronary Artery Disease

Presentation: A 48-year-old female from Jharkhand with a history of hypothyroidism and prior coronary artery disease (CAD), status post percutaneous coronary intervention to right coronary artery (RCA) a year back, presented with subacute onset of heart failure symptoms: two months of progressive exertional dyspnoea (New York Heart Association (NYHA) class III) and one week of new-onset effort angina (Canadian Cardiovascular Society (CCS) class II) and pedal oedema. Notably, her past echocardiogram a year ago had shown only mild valvular lesions: mild AS and mild MR.

**Figure 1:**
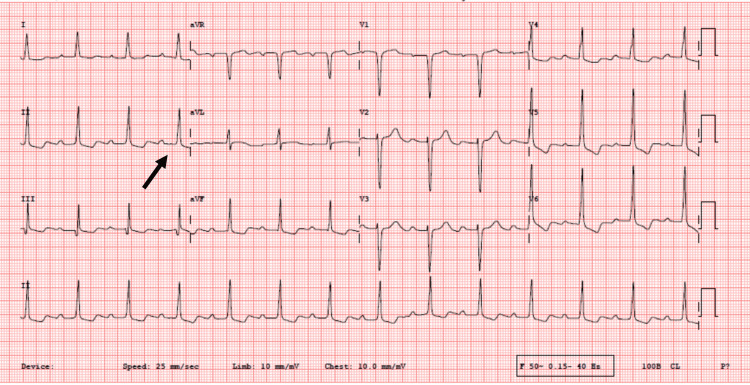
Case 2: Electrocardiogram (ECG) ECG showing the first-degree atrioventricular block (black arrow).

Clinical and diagnostic findings: On examination, the patient was febrile. Cardiac auscultation revealed an early diastolic murmur and an ejection systolic murmur in the aortic area. The ECG was significant for first-degree atrioventricular block (PR interval >200 ms) (Figure 1), a minor manifestation of ARF. Blood cultures failed to demonstrate any significant bacterial growth.

**Figure 2:**
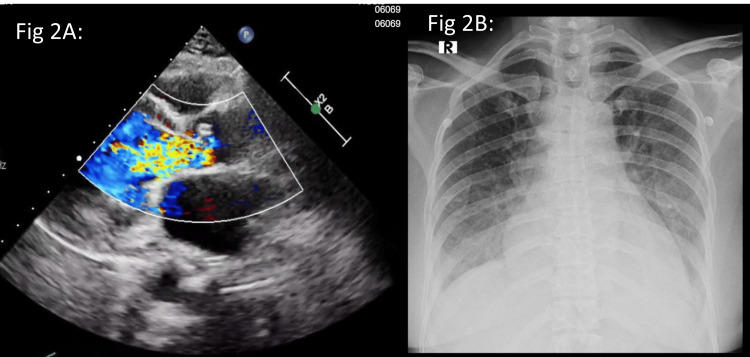
Case 2: Transthoracic echo and chest X-ray Transthoracic echo (2A) colour Doppler image showing flow turbulence across the aortic valve, suggestive of aortic regurgitation. Chest X-ray (2B) showing cardiomegaly with pulmonary venous congestion.

Imaging and laboratory: Her current echo demonstrated a rapid progression of valvular disease, showing severe aortic regurgitation (AR) and moderate AS, with preserved left ventricular function, and chest X-ray (CXR) showed cardiomegaly with pulmonary venous congestion (Figure 2). Coronary angiogram revealed a patent stent in the RCA, and the left system was also normal. Laboratory studies confirmed an acute inflammatory process with elevated CRP (6.22 mg/L) and ESR (65 mm in one hour). Streptococcal serology was significantly elevated (ASO: 1000 IU/mL and ADNB: 611 U/mL) (Table 1).

Diagnosis and management: The clinical presentation of acute worsening carditis (new onset of severe AR and AS), combined with first-degree AV block and clear serological evidence of recent streptococcal infection, confirmed the diagnosis of ARF reactivation. Ischemic aetiology of valvular progression was ruled out, as coronary angiography did not show obstructive CAD, and her age was against a degenerative condition. Initial management focused on optimising heart failure symptoms with diuretics and non-invasive ventilation. Given the active carditis, the patient was started on oral steroids (prednisolone 0.5 mg/kg/day). Surgical intervention for the severe valvular lesions was deferred to allow the anti-inflammatory therapy to take effect, with plans for reassessment post-steroid taper.

Case 3

Atypical Presentation Complicated by Suspected Infective Endocarditis

Presentation: A 43-year-old male from Jharkhand presented with a complex, two-month history of intermittent low-grade fever, cough, abdominal discomfort, and polyarthralgia. This progressed over one month to symptoms of heart failure, including retrosternal chest pain (exacerbated by inspiration, suggestive of pericarditis), progressive dyspnoea (NYHA class II to IV), and pedal oedema.

Clinical findings and initial evaluation: Examination revealed fever, pallor, pedal oedema, clubbing, and elevated jugular venous pressure. A generalised purpuric rash and petechiae were noted, along with splenomegaly (2 cm below the left costal margin). On auscultation pan-systolic murmur was heard in the mitral area and an early diastolic murmur in the aortic area.

The differential diagnosis for this presentation of pyrexia of unknown origin, purpuric rash, polyarthralgia, and valvular heart failure (MR and AR) encompassed infective endocarditis, medium-vessel vasculitis, malignancy, and systemic infections, specifically brucellosis, melioidosis, and tuberculosis. Blood workup for infections and autoimmune causes was negative. Transthoracic echocardiogram showed RHD changes (doming mitral leaflets), moderate MR/AR, mild MS, and a mild pericardial effusion.

**Figure 3:**
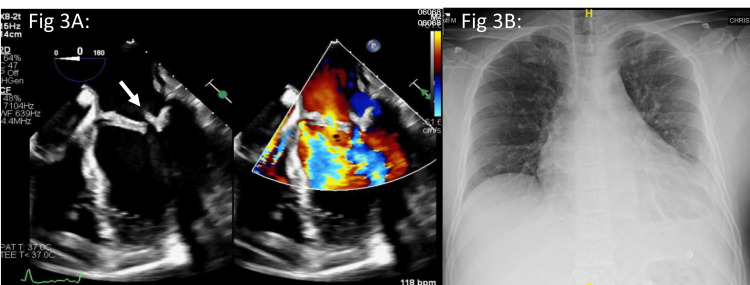
Case 3: Transesophageal echo and chest X-ray Transoesophageal echo (3A) showing an echogenic nodule at the tip of the posterior mitral leaflet on the atrial aspect (white arrow). Chest X-ray (3B) showing cardiomegaly and mild left pleural effusion.

Diagnostic dilemma and intervention: Transoesophageal echocardiography (TEE) confirmed RHD changes and revealed an echogenic mobile structure on the atrial aspect of the posterior mitral leaflet, suggestive of a vegetation (Figure 3). CXR showed cardiomegaly and left pleural effusion (Figure 3). Based on the echo findings (major criteria) and fever (minor criteria), the patient was empirically treated for culture-negative IE with ceftriaxone and vancomycin for six weeks. Post-antibiotic course (after 6 weeks), he was taken up for valve replacement surgery. Once midline sternotomy was performed, it was noticed that he had dense, inflamed, and extensive pericardial adhesions. In view of the inflamed myocardium and pericardium, the surgery was abandoned.

Final diagnosis and management: Post-operatively, suspicion of active rheumatic disease was re-raised by the surgical findings (inflamed pericardium). Streptococcal titres were sent, and not to our surprise, both were elevated (ASO: 353 IU/mL and ADNB: 1030 U/mL) (Table 1). The clinical picture, combined with the surgical findings and elevated antistreptococcal titres, confirmed a diagnosis of acute rheumatic reactivation with severe carditis (as per the modified Jones criteria). The presumed vegetation was attributed to a rheumatic nodule. The histopathology of the pericardial tissue revealed fibrous tissue with no granuloma. The patient was started on a tapering course of oral steroids and aspirin. A follow-up echo showed resolution of the pericardial effusion, and he was scheduled for definitive valvular surgery after completion of the anti-inflammatory course.

Case 4

Post-double Valve Replacement Pericardial Effusion Due to Rheumatic Recurrence

**Figure 4:**
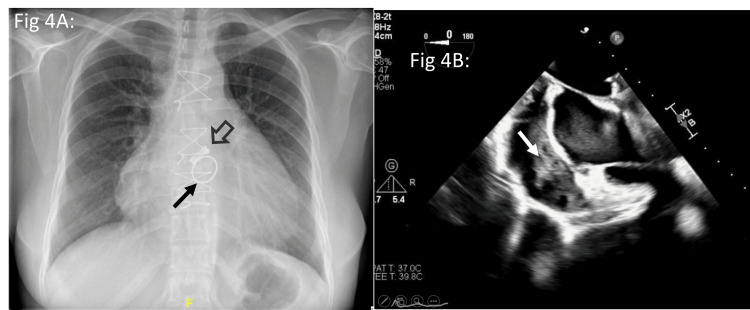
Case 4: Chest X-ray and transoesophageal echo Chest X-ray (4A) showing cardiomegaly, prosthetic aortic (open arrow) and mitral (solid arrow) valves. Transoesophageal echo (4B) showing organised pericardial collection (white arrow).

Presentation: A 43-year-old lady from Jharkhand presented with a history of recurrent fever for the past two months. She was apparently in her usual state of health until a year back, when she developed right-sided hemiparesis. During evaluation, she was detected to have RHD. She then underwent double valve replacement (DVR) (aortic: 19 mm bicarbon slimline valve and mitral: 25 mm SJM valve) (Figure 4). She was alright till 3 months post-surgery, then she developed recurrent episodes of fever. She was worked up for IE, which was negative. ESR and CRP were raised. She was diagnosed with urosepsis; however, the urine culture was negative. When she followed up in another hospital, she was given a course of antibiotics as the throat culture was positive for *Streptococcus pneumoniae*.

Clinical and diagnostic workup: On presentation, she was febrile, with an otherwise unremarkable examination. Her blood work showed an increase in inflammatory markers. Her serology showed ASO 1030 IU/mL and ADNB 799 U/mL (Table 1). Her transthoracic echo was suggestive of a large pericardial effusion (maximal depth of 20 mm). A TEE was performed, which showed normally functioning prosthetic valves. A loculated pericardial effusion was noted in the right atrial aspect, which appeared organised with strands; there were no vegetations, effectively ruling out prosthetic valve IE as the cause of the effusion/fever (Figure 4).

Final diagnosis and treatment: Pericardiocentesis was performed, yielding haemorrhagic, exudative fluid that was sterile and negative for malignant cells. Given the exclusion of infection, the combination of recurrent fever, inflammatory pericarditis (a major manifestation of ARF carditis), and elevated streptococcal titres (ASO: 1030 IU/mL and ADNB: 799 U/mL) was diagnostic of ARF Recurrence. Also, post-surgery, she did not receive her regular dose of penicillin for the last six months, predisposing her to this recurrence. Another close differential, post-pericardiotomy syndrome (PPS), was ruled out, as PPS occurs in the first three months of surgery [10]. She was initiated on a course of aspirin (0.5 mg/kg) for the carditis and promptly started on long-term secondary penicillin prophylaxis. Her fever and symptoms resolved completely, and she remained symptomatically better on follow-up.

Case 5

Breakthrough Recurrence While on Prophylaxis

Presentation: A 33-year-old female from Tamil Nadu presented with a one-month history of low-grade fever and multiple large joint pain. Two years prior, she was diagnosed with ARF accompanied by carditis and moderate MR; she was on secondary prophylaxis with benzathine penicillin every 21 days. She also noted new skin lesions over the extensor aspect of arms and elbows, which were diagnosed as psoriasis by dermatology.

Clinical findings: Systemic examination was unremarkable for new carditis. Differentials considered were rheumatic reactivation and psoriatic arthritis.

Laboratory and diagnosis: Inflammatory markers were elevated. Streptococcal titres were significantly elevated despite being on prophylaxis (ASO: 1710 IU/mL and ADNB: 4040 U/mL) (Table 1). The presence of polyarthritis and significantly elevated titres while on prophylaxis confirmed a diagnosis of ARF reactivation.

Management and outcome: She was treated with naproxen, which led to a significant improvement in her arthritic symptoms. Given the breakthrough recurrence despite being strictly adherent to once in three weekly regimen, she was counselled and advised a stricter regimen of benzathine penicillin prophylaxis every two weeks to prevent further episodes. Though there are no guidelines for once in two weeks prophylaxis, we started her in line with some previous studies where the results have been better [3,11].

Case 6

Silent Arthritis, Severe Carditis: ARF Presenting as Decompensated Heart Failure and Pericardial Effusion

Presentation: A 34-year-old gentleman from Vellore, Tamil Nadu, presented with a one-week history of gradually progressive dyspnoea and orthopnoea, with no history of fever, joint pain, or cough. He had chronic non-healing ulcers on his gluteal region and left foot. He had no history of diabetes mellitus or hypertension.

Clinical findings: Examination revealed a soft S1 with an apical mid-diastolic murmur and a short systolic murmur. Surgical consultation was sought for the ulcers, which did not appear to be the primary cause of his systemic illness. Echocardiography was critical, demonstrating severe MS (mean pressure gradient: 11 mmHg and mitral valve area: 0.81 cm^2^), moderate MR, severe tricuspid regurgitation, severe left and right ventricular dysfunction, and a moderate pericardial effusion (maximal depth: 23 mm).

Laboratory and diagnosis: Given the presentation of new-onset heart failure and pericardial effusion, rheumatic carditis was suspected. Streptococcal titres were sent; while ASO was normal (144 IU/mL), ADNB was significantly elevated (2080 U/mL) (Table 2), fulfilling the major criterion (pericarditis/carditis) and evidence of recent infection.

**Table 2:** Laboratory results ADNB, anti-DNase B; ASO, anti-streptolysin O; CRP, C-reactive protein; ESR, erythrocyte sedimentation rate.

Case no	Age	Sex	ESR ref: Male: 3-10 mm/hr, female: 5-20 mm/hr	CRP ref: <6 mg/L	ASO ref: <300 IU/mL	ADNB ref: <500 U/mL
1	37	F	120	46	1520	2800
2	48	F	65	6.88	1000	611
3	43	M	149	75	353	1030
4	43	F	20	2.66	1030	799
5	33	F	56	33.1	1710	4040
6	34	M	109	10.6	144	2080

Management and outcome: The patient was diagnosed with acute rheumatic carditis and promptly started on systemic steroids and intramuscular benzathine penicillin for secondary prophylaxis. His anti-failure medications were optimised. He was discharged on a tapering steroid regimen and was planned to transition to aspirin once inflammatory markers subsided, with a plan for continued long-term penicillin prophylaxis.

## Discussion

Re-evaluating the age paradigm and epidemiology

Though rheumatic fever or RHD has practically disappeared in the developed world, in developing countries like India, it is a major health concern, primarily in children and the young population. Based on population-based studies in the world, the incidence of acute RF varies from 5 to 51/100,000 population with a mean of 19/100,000 [12]. The annual incidence of ARF is between 100 and 200 per 1,00,000 children of school age, and that of RHD is around 600,000 children in India [8]. Padmavati et al. reported that the average age at presentation in India is between 10 and 14 years [13].

The classical understanding posits ARF as a disease of children and adolescents, with the incidence sharply declining after age 25 years. First episodes of ARF are most common just before adolescence, wane by the end of the second decade, and are rare in adults aged >35 years. Recurrent episodes are especially frequent in adolescence and early adulthood, and occasional cases are observed in people aged >45 years [14]. The presence of very few studies on rheumatic fever in adults also points towards the condition’s rarity in this age group [15-18].

ARF is a multifactorial disease that follows group A streptococcal (GAS) (the agent) pharyngitis in a susceptible individual (the host) who lives under deprived social conditions (the environment). The autoimmune pathogenesis of ARF in adults is similar to paediatric ARF. Molecular mimicry theory explains how GAS pharyngitis can cause an autoimmune response in certain individuals, leading to cross-reactivity with similar epitopes in various organs, including the heart, brain, joints, and skin. If epidemic GAS pharyngitis goes untreated, up to 3% of patients may develop the disease, though effective antibiotic therapy drastically reduces this risk [19].

Frequent GAS infections in the throat and skin contribute to a breakdown in immune tolerance through molecular mimicry. This process induces epitope spreading, causing the immune response to increasingly target cardiac myosin and various other proteins within the heart. This explains that recurrent ARF causes more damage to cardiac valves, resulting in RHD [20].

Our case series, presenting six instances of ARF episode/recurrence in adults aged >30 years (three patients with first episodes of ARF and three patients with ARF reactivation), challenges this age paradigm. While first episodes remain rare in this age group, the clustered presentation of recurrence in middle-aged adults (34-48 years) underscores that ARF remains a relevant, and often overlooked, differential diagnosis in adult cardiology settings within endemic areas. This pattern suggests that the adult population continues to bear the risk of recurrence, particularly when prophylaxis is interrupted or inadequate.

Atypical presentation and diagnostic pitfalls

The cases highlight the highly variable and often subtle manifestations of ARF in adults, demanding a low diagnostic threshold from clinicians.

Isolated Carditis

Case 6 is a striking example of isolated carditis presenting as severe, decompensated heart failure and pericardial effusion, entirely lacking the classic symptoms of fever or polyarthritis. This "silent" presentation makes diagnosis challenging and reliant on high inflammatory markers and serology.

The IE Mimic

Case 3 illustrates a critical diagnostic pitfall. The patient presented with fever, heart failure, and a suspected valvular vegetation on TEE, initially leading to treatment for IE. The final diagnosis of ARF was supported by the surgical finding of dense inflammatory pericardial adhesions and significantly elevated streptococcal titres. This finding suggests that the "vegetation" was likely an acute rheumatic nodule, reinforcing the need for simultaneous serology in suspected patients. IE is one of the mimics of ARF in adults, considering the common clinical features of fever, valve involvement, and vegetation/nodule [21]. In patients with IE-ARF overlap symptoms, it is recommended to perform the dual streptococcal serology to aid in confirming the diagnosis.

Serology Reliance

The diagnostic utility of dual serology is demonstrated by Case 6, where the ASO was normal, but the ADNB was significantly elevated. This stresses the need to check both ASO and ADNB titres, especially when carditis is suspected, as one may be negative while the other confirms a recent streptococcal infection.

The critical role of prophylaxis: adherence and intensity

The recurrence of ARF in these adult patients emphasises key failures in secondary prevention:

Non-adherence

Post-DVR, Cases 1 and 4 demonstrate that non-adherence to secondary penicillin prophylaxis remains the primary driver of recurrence, irrespective of the patient's age or complexity of underlying disease.

Breakthrough Recurrence

Case 5 is a crucial example of a breakthrough recurrence occurring despite the patient being on the standard benzathine penicillin regimen. This outcome indicates that in high-risk patients, especially those in highly endemic regions, the standard regimen may be insufficient. A stricter regimen (e.g., every 14 days) may be required to maintain adequate serum penicillin levels and ensure effective secondary prevention [3,11]. The generalisability of these findings requires further large-scale studies focusing on recurrence rates, particularly as a twice-weekly regimen incurs higher costs, increased patient discomfort, and greater resource utilisation.

Unlike certain other common diseases of childhood, rheumatic fever leaves no known immunity and seems to create an increased susceptibility to recurrent attacks. The effects of these recurrent attacks have been unpredictable. Numerous studies have shown that patients presenting with isolated arthritis or chorea tend to experience recurrences with similar clinical phenotypes, often sparing the heart in subsequent episodes. Although this repetitive pattern predominated, it was not universal. Other patients seemed to escape heart disease with their first episode but acquired evidence of it at later attacks. In another group, heart disease occurred at the first rheumatic attack, and the recurrences often made it worse [22]. In patients with previous ARF, the risk of recurrence is more than 50%.

Certain risk factors for recurrence include socioeconomic factors such as overcrowding, poor accessibility to health care, risk of exposure to GAS, and non-compliance with penicillin. Regarding host factors, human leukocyte antigen (HLA) class II alleles have been associated with ARF and RHD around the world; the DR7 allele is the most frequently associated with the condition. In addition, the class I HLA allele HLA*B5 is associated with developing ARF and the formation of immune complexes during ARF [20].

When reactivation happens, there is more valvular damage and worsening. It is important to prevent recurrences by proper secondary prophylaxis and to recognise the recurrences and treat them appropriately.

According to the World Health Organization (WHO) guidelines, secondary prophylaxis should continue for at least five years after the last episode of ARF or until the age of 18 years (whichever is longer) and for a greater length of time in case of carditis or RHD.

Secondary prophylaxis with regular intramuscular injection of benzathine penicillin G (BPG) is a key component of ARF and RHD control programs. The WHO recommends three to four weekly BPG continued for a duration dependent on factors, including age, time since the last episode, risk of streptococcal infections in the area, and presence of RHD [23].

In a study by Sarkar et al. in Madhya Pradesh, they found that out of 100 patients of RHD, 38 had a hospital visit for rheumatic reactivation. Out of 38 patients, 32 patients were not taking prophylaxis, suggesting 80% patients were unprotected. This further reinstates the importance of secondary penicillin prophylaxis [24]. Proper adherence to secondary prophylaxis prevents recurrences in patients who had previous ARF, and it is a key factor for reducing the burden of RHD in endemic regions [25-28]. Factors which affect adherence are the non-availability of penicillin in certain states in India [29] and poor counselling about the importance of adherence [30].

There should be high focus on proper counselling in health care system about penicillin prophylaxis to ARF patients, and the availability should be uninterrupted. A study assessed adherence to penicillin prophylaxis during the COVID-19 pandemic in a tertiary care hospital and reported an 80% adherence, with non-availability as one reason affecting adherence [31].

Thus, we highlight the varied and sometimes unusual presentations of ARF in adults and aim to stress a key clinical message: Physicians must maintain a low diagnostic threshold for ARF, irrespective of the patient's age. This study further underscores the critical importance of strict adherence to secondary penicillin prophylaxis to prevent further recurrence and mitigate the long-term risk of severe chronic valvular heart disease.

## Conclusions

In summary, this case series demonstrates that rheumatic fever and its recurrence remain significant pathologies in the adult age group in our setting. Clinicians must maintain a low index of suspicion for ARF in adults presenting with fever, joint pain, or new/worsening cardiac symptoms, especially when ruling out other aetiologies. Furthermore, these cases forcefully reinforce the necessity of both strict adherence to prophylaxis and, in selected high-risk patients, potentially intensifying the prophylaxis regimen to mitigate the severe long-term burden of chronic valvular heart disease. We accept our case series involves small single centre data and for practice change especially intensifying prophylaxis regimen we might require future larger studies and registry based studies to validate such a recommendation in adult population.
